# Atypical Presentation of Toxic Shock Syndrome in a Patient With Postpartum Necrotizing Endometritis

**DOI:** 10.7759/cureus.94106

**Published:** 2025-10-08

**Authors:** Tamar Megrelishvili, Elene Saribekovi, Elene Pachkoria, Tamar Didbaridze, Ia Mikadze, Levan Ratiani

**Affiliations:** 1 Department of Infectious Diseases, First University Clinic of Tbilisi State Medical University, Tbilisi, GEO; 2 Department of Infectious Diseases, Tbilisi State Medical University, Tbilisi, GEO; 3 Department of Microbiology, First University Clinic of Tbilisi State Medical University, Tbilisi, GEO; 4 Department of Anesthesiology, First University Clinic of Tbilisi State Medical University, Tbilisi, GEO

**Keywords:** cesarean section, immunothrombosis, postpartum endometritis, prophylactic anticoagulation, toxic shock syndrome (tss)

## Abstract

A 42-year-old woman with a history of two first-trimester miscarriages and a known prothrombotic polymorphism (MTHFR C677T/A1298C compound heterozygote) developed postpartum toxic shock syndrome (TSS) complicated by peripheral skin necrosis eight days after emergency cesarean section for preeclampsia exacerbation. She presented with fever, hypotension, tachycardia, severe diarrhea, and a purpuric rash on the nose and distal lower extremities, rapidly evolving to necrosis. Although she had been prescribed prophylactic nadroparin during pregnancy, anticoagulation was discontinued upon discharge following the cesarean section. Laboratory findings revealed markedly elevated inflammatory markers, mild coagulopathy, and acute kidney injury. Coagulation abnormalities were mild and did not meet criteria for disseminated intravascular coagulation. On the second day of hospitalization, a hysterectomy was performed, and histopathology confirmed necrotizing endometritis. With early surgical source control, broad-spectrum antibiotics, anticoagulation, and intensive supportive care, the patient’s condition improved significantly. Skin lesions gradually stabilized with desquamation and partial healing by day 14. This case highlights an unusual dermatologic manifestation of TSS and underscores the potential contribution of MTHFR C677T/A1298C polymorphisms to thromboinflammatory complications in the postpartum period.

## Introduction

Toxic shock syndrome (TSS) is a rare but potentially life-threatening condition caused by exotoxin-producing strains of *Staphylococcus aureus* or *Streptococcus pyogenes*. It is characterized by the sudden onset of fever, hypotension, multiorgan dysfunction, and a diffuse rash, often followed by desquamation. Although classically associated with tampon use, TSS can also occur in soft tissue infections, post-surgical infections, burns, retained foreign bodies such as nasal packing, and dialysis catheters [1].

The toxins function as superantigens, triggering an abnormal immune response by bypassing the usual antigen-specific activation of T cells by antigen-presenting cells (APCs). Instead, they cause widespread, nonspecific activation of 5%-30% of all T cells, leading to an overwhelming release of proinflammatory cytokines [2].

Staphylococcal TSS is primarily a clinical diagnosis; culture results may support the diagnosis but are not required. In contrast, a positive culture from a sterile site is essential to confirm the diagnosis of streptococcal TSS [3].

Immunothrombosis, originally described in the context of sepsis, is a host defense mechanism that integrates the actions of innate immune cells, platelets, and the coagulation system to trap and eliminate pathogens through localized fibrin clot formation. Although this process is protective in early infection, it can become dysregulated during systemic inflammation, as seen in TSS, leading to widespread microvascular thrombosis, coagulopathy, and organ dysfunction [4].

Methylenetetrahydrofolate reductase (MTHFR) is a critical enzyme involved in the folate-dependent metabolism of homocysteine to methionine. Genetic polymorphisms in the MTHFR gene, notably the C677T and A1298C variants, are associated with reduced enzymatic activity, which can result in elevated plasma homocysteine levels (hyperhomocysteinemia). Although hyperhomocysteinemia has long been associated with venous thromboembolism (VTE), plasma homocysteine levels do not consistently correlate with MTHFR polymorphisms nor independently predict thrombotic risk. Certain MTHFR gene variants-particularly compound heterozygosity for C677T and A1298C-have been reported to modestly increase VTE risk in some studies. Therefore, genotyping for MTHFR mutations may provide a more stable genetic basis for risk assessment in selected clinical settings. When evaluated alongside other clinical factors, this information can support individualized risk stratification and inform consideration of prophylactic measures for VTE prevention [5].

In a study of 200 Palestinian women (100 with unexplained recurrent miscarriage and 100 controls), the analysis of eight inherited thrombophilia mutations revealed a significantly higher frequency of the MTHFR A1298C mutant allele in patients with unexplained recurrent miscarriage compared to controls [6]. We present the case of a 42-year-old woman MTHFR C677T/A1298C compound heterozygote, who developed postpartum TSS complicated by peripheral skin necrosis after an emergency cesarean section. The patient’s obstetric history, genetic background, and rapid clinical deterioration highlight the need for extended vigilance and individualized postpartum thromboprophylaxis in women at elevated risk for thromboinflammatory complications.

## Case presentation

**Figure 1:**
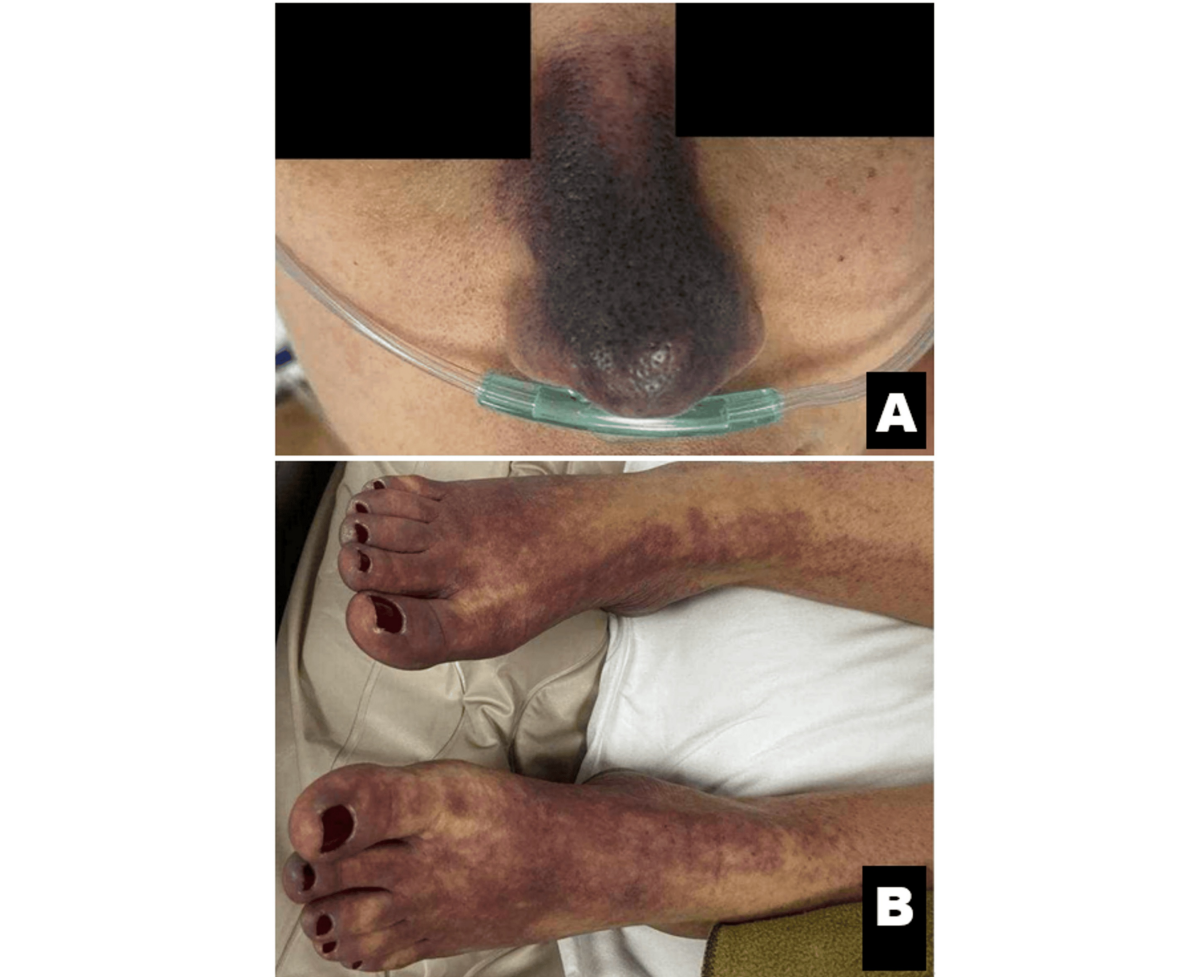
Purpuric-necrotic rash on the nose (A) and distal lower extremities (B) on the day of admission.

A 42-year-old female patient, gravida 3, para 1, presented to the hospital with a one-day history of severe diarrhea, fever, chills, profound weakness, and a newly developed purpuric rash localized to the nose and distal lower extremities (Figure 1). Eight days prior, she had undergone an emergency cesarean section at 38 weeks of gestation due to exacerbation of preeclampsia. Her obstetric history was notable for first-trimester miscarriages, which led to genetic testing revealing MTHFR C677T/A1298C compound heterozygosity. During pregnancy, she received prophylactic nadroparin (0.3 mL/day) until hospital discharge after cesarean, which she then discontinued without starting alternative anticoagulation therapy.

The patient was admitted to the intensive care unit (ICU) for close monitoring and management. A multidisciplinary team including obstetrics, infectious diseases, and critical care specialists was involved in the patient’s care.

On admission, the patient was hypotensive (blood pressure (BP) 70/50 mmHg), tachycardic (heart rate (HR) 132 bpm), and febrile (39°C). She was alert and oriented but displayed severe generalized weakness and adynamia, without focal neurological deficits. Due to hemodynamic instability, vasopressor support was promptly initiated, stabilizing her BP to 113/65 mmHg. The Sequential Organ Failure Assessment (SOFA) score was 9, indicating significant organ dysfunction.

**Figure 2:**
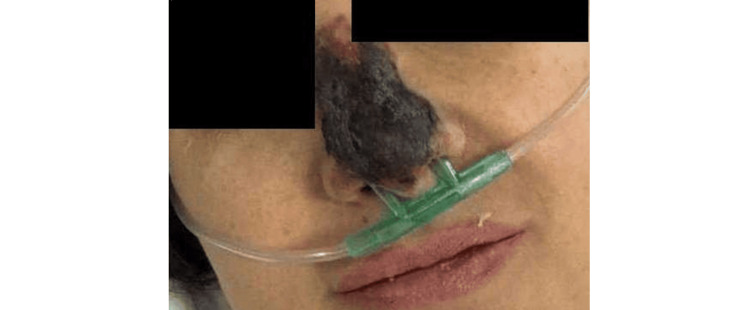
Sharply demarcated skin necrosis of the nose, observed on the third day of admission.

Physical examination revealed pallor and impaired distal microcirculation in the lower extremities. A purpuric rash was evident on the nose and distal lower limbs, which rapidly progressed to sharply demarcated skin necrosis by the third day of admission (Figure 2).

The cesarean incision was healing well without signs of local infection. Laboratory investigations showed markedly elevated C-reactive protein (CRP) and leukocytosis with neutrophilia, alongside severe lymphopenia (Table 1). Liver function tests showed mild elevation, whereas urea and creatinine levels were significantly elevated (Table 2). Coagulation studies showed prolonged prothrombin time, decreased prothrombin index, elevated international normalized ratio (INR), and mildly reduced fibrinogen levels, while activated partial thromboplastin time (aPTT) remained within normal limits (Table 3).

**Table 1:** Laboratory analysis results before hysterectomy. WBC: white blood cell; Lymph: lymphocytes; Neu: neutrophils; RBC: red blood cells; HGB: hemoglobin; HCT: hematocrit; PLT: platelets; CRP: C-reactive protein

Parameter	Result	Reference range
WBC (10^9^/L)	20.32	4.00-11.00
Lymph (%)	1.8	20.0-45.0
Lymph (10^9^/L)	0.37	1.00-5.00
Neu (%)	96.30	50.00-70.00
Neu (10⁹/L)	19.56	2.00-7.00
Bands (%)	3.70	0.01-1.50
Bands (10⁹/L)	0.17	0.01-0.20
RBC (10¹²/L)	3.85	3.80-5.40
HGB (g/dL)	11.6	12.5-15.5
HCT (L/L)	34.7	36.0-45.0
PLT (10³/µL)	211	150-380
CRP (mg/L)	174	<5
Procalcitonin (ng/mL)	>10	<0.5

**Table 2:** Biochemistry results on admission. AST: aspartate aminotransferase; ALT: alanine aminotransferase

Parameter	Result	Reference range
Urea (mmol/L)	11.45	2.76-8.07
Creatinine (µmol/L)	344.7	45.00-84.00
AST (U/L)	64	≤32
ALT (U/L)	48	≤35

**Table 3:** Coagulation profile on admission. aPTT: activated partial thromboplastin time; PT: prothrombin time; INR: international normalized ratio; PI: prothrombin index

Parameter	Result	Reference range
aPTT (seconds)	39.4	25.4-43
PT (seconds)	18.0	12.1-16.5
INR	1.57	0.9-1.3
PI (%)	54	76-104
Fibrinogen (g/L)	1.9	2-4

Broad-spectrum antibiotic therapy with meropenem, vancomycin, and moxifloxacin was initiated to cover Gram-positive, Gram-negative, and anaerobic organisms, selected with consideration of local resistance patterns, and adjusted to the patient's creatinine clearance. Additionally, hormonal treatment (dexamethasone 4 mg intravenously three times daily), gastroprotective therapy (pantoprazole 40 mg intravenously once), and anticoagulation therapy (nadroparin 0.4 mL subcutaneously once daily) were commenced. Blood cultures were repeatedly negative. Despite the absence of identifiable bacteremia, the patient exhibited classic features of toxin-mediated systemic illness, including severe diarrhea, hypotension, high fever, and multiorgan dysfunction, consistent with a diagnosis of TSS.

**Figure 3:**
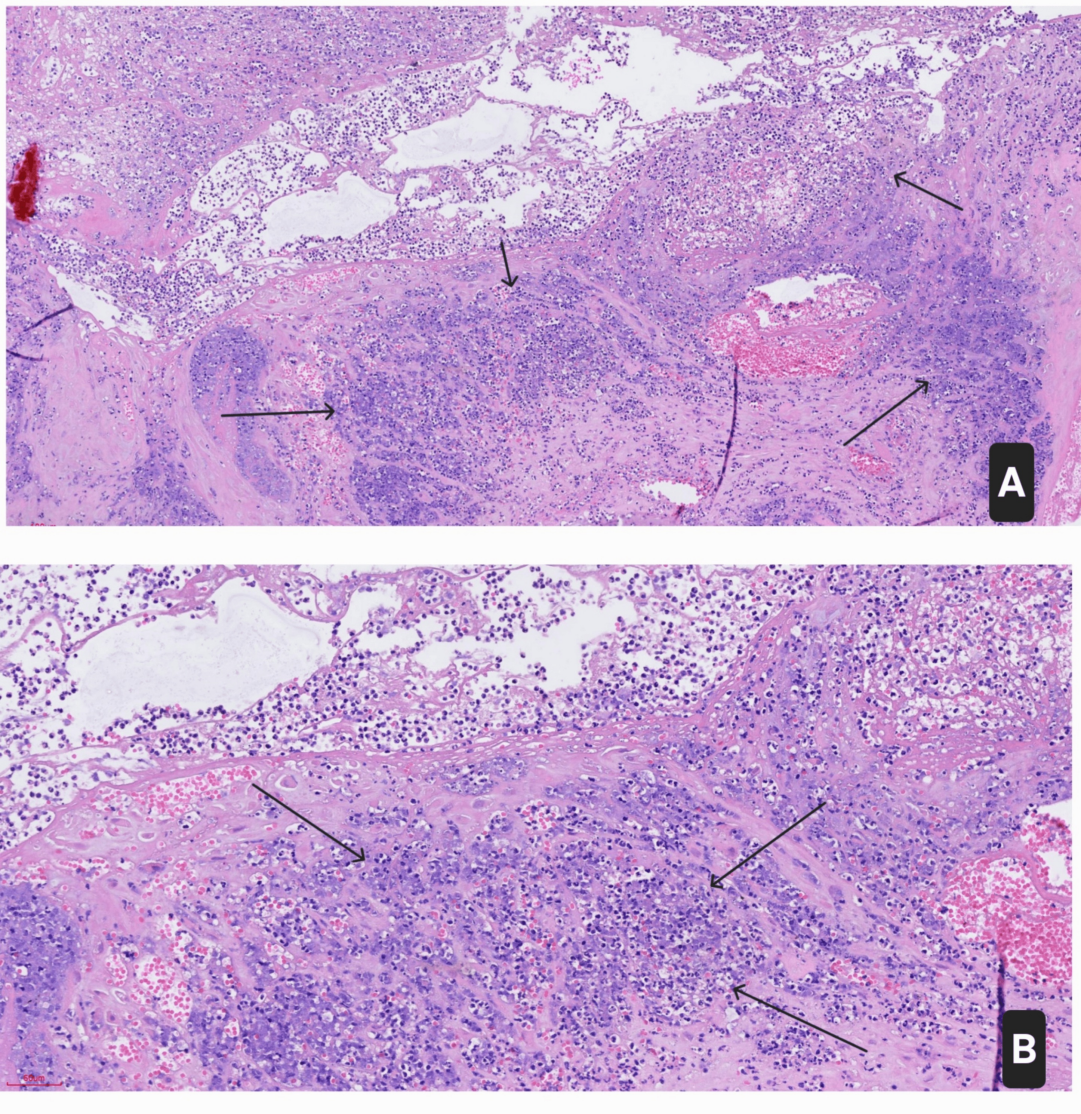
Histopathology of acute necrotizing endometritis (H&E, (A) 100×; (B) 200×) showing extensive endomyometrial destruction, tissue necrosis, and dense acute inflammatory infiltrates (black arrows). H&E: hematoxylin and eosin

Due to the severity and progression of the clinical state, a hysterectomy was performed on day two to control the suspected source of toxin production. The decision was made collaboratively, taking into account the patient’s preferences and her completed family planning. Histopathology confirmed acute necrotizing endometritis, with extensive tissue destruction and inflammation (Figure 3).

**Figure 4:**
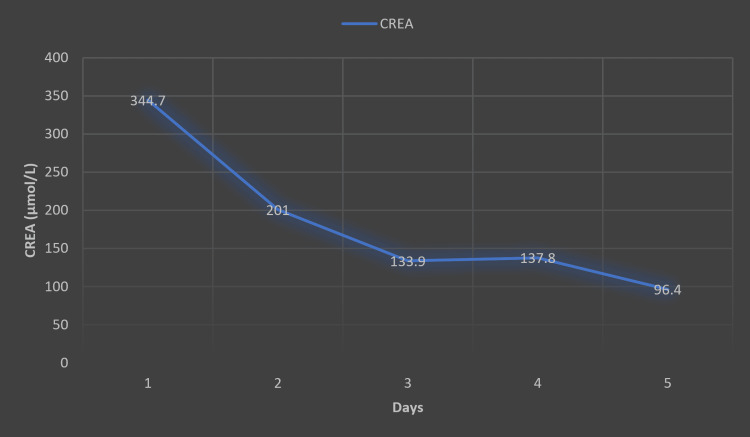
Declining CREA levels from day 1 to day 5 of admission. Reference range: CREA: 45.00-84.00 µmol/L. CREA: creatinine

**Figure 5:**
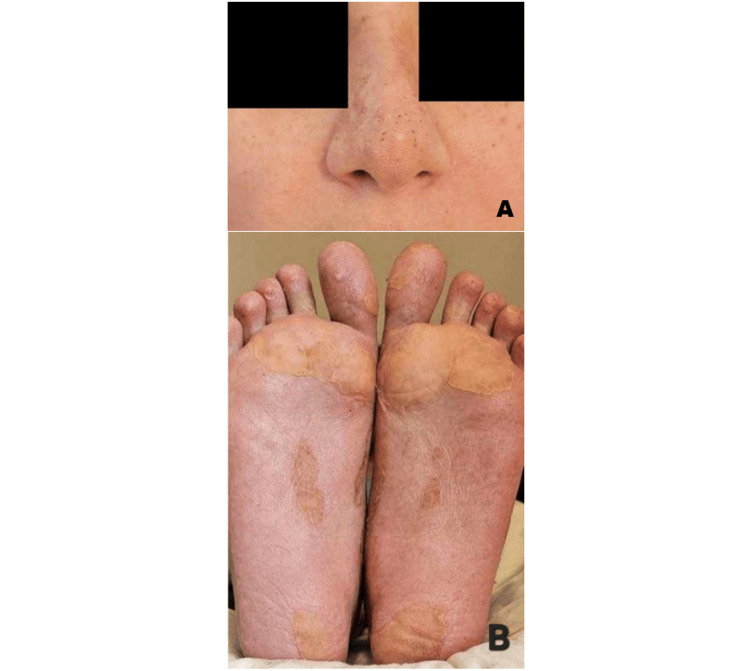
(A, B) All lesions significantly improved, with prominent desquamation observed on the 14th day of admission.

The clinical course demonstrated significant improvement following the hysterectomy. Laboratory tests showed a reduction in leukocyte count and CRP levels, although both remained significantly elevated, along with procalcitonin and D-dimer (Table 4). On the 10th day of admission, laboratory findings were within the reference range, except for slightly elevated CRP and D-dimer (Table 5). Creatinine levels progressively decreased (Figure 4). The skin necrosis stabilized and progressively healed, with prominent desquamation observed (Figure 5). By the 14th day of admission, the patient was discharged in a stable condition.

**Table 4:** Laboratory analysis results after hysterectomy. WBC: white blood cell; Lymph: lymphocytes; Neu: neutrophils; CRP: C-reactive protein

Parameter	Result	Reference range
WBC (10^9^/L)	12.95	4.00-11.00
Lymph (%)	6.8	20.0-45.0
Lymph (10⁹/L)	0.88	1.00-5.00
Neu (%)	89.6	50.00-70.00
Neu (10⁹/L)	11.6	2.00-7.00
Bands (%)	1.8	0.01-1.50
Bands (10⁹/L)	0.23	0.01-0.20
CRP (mg/L)	137.8	<5
Procalcitonin (ng/mL)	>10	<0.5
D-dimer (mg/L)	>10	0.1-0.50

**Table 5:** Laboratory analysis results showing significant improvement on the 10th day of admission. WBC: white blood cell; Lymph: lymphocytes; Neu: neutrophils; CRP: C-reactive protein

Parameter	Result	Reference range
WBC (109/L)	8.79	4.00-11.00
Lymph (%)	31.3	20.0-45.0
Lymph (10⁹/L)	2.75	1.00-5.00
Neu (%)	63.4	50.00-70.00
Neu (10⁹/L)	5.57	2.00-7.00
Bands (%)	0.5	0.01-1.50
Bands (10⁹/L)	0.04	0.01-0.20
CRP (mg/L)	9	<5
Procalcitonin (ng/mL)	<0.1	<0.5
D-dimer (mg/L)	2.95	0.1-0.50

## Discussion

This case illustrates a rare and complex presentation of postpartum TSS complicated by purpura fulminans-like skin lesions in a patient with MTHFR C677T/A1298C compound heterozygosity. Although streptococcal TSS was considered, the absence of group A *Streptococcus* in cultures, combined with the clinical presentation, suggests a staphylococcal etiology.

While TSS is classically characterized by the sudden onset of high fever, hypotension, rash, and multiorgan involvement, the dermatologic findings in this patient did not resemble the diffuse erythematous rash typical of TSS [7]. The presence of sharply demarcated skin necrosis, particularly in distal extremities and the nasal tip, supports the notion of localized thrombotic events in the setting of systemic inflammation-a phenomenon often described in purpura fulminans [8] or catastrophic antiphospholipid syndrome (CAPS) [9], though this patient had no clinical or serologic evidence for either. Nonetheless, this dermatologic manifestation in the context of infection and thrombophilia underscores the diagnostic challenge.

Liver function tests demonstrated mild abnormalities, with aspartate aminotransferase (AST) elevated to approximately twice the upper limit of normal, meeting the threshold commonly used in TSS diagnostic criteria. However, alanine aminotransferase (ALT) remained below this twofold cutoff, making the hepatic involvement less pronounced than is typically observed in classic cases of TSS, where both AST and ALT are often markedly elevated. The coagulation profile demonstrated only mild abnormalities, including prolonged prothrombin time, elevated INR, decreased prothrombin index, and slightly reduced fibrinogen, while aPTT remained normal. This mild coagulopathy distinguishes the case from classic disseminated intravascular coagulation (DIC), which usually features more severe derangements [10].

Empiric antimicrobial therapy with meropenem, vancomycin, and moxifloxacin was initiated to ensure broad-spectrum coverage, including Gram-positive, Gram-negative, and anaerobic organisms. This regimen was selected based on local antimicrobial resistance patterns and clinical suspicion of polymicrobial postpartum endometritis rather than classical TSS. Clindamycin, typically used in endometritis and TSS due to its ability to inhibit toxin production, was not included because of high regional resistance rates. Given the patient's rapid clinical improvement following hysterectomy and supportive care, no changes to the initial antibiotic regimen were necessary, and adjunctive therapies such as intravenous immunoglobulin (IVIG) were not pursued.

MTHFR C677T and A1298C polymorphisms lead to elevated levels of total homocysteine and are associated with recurrent adverse obstetrical outcomes [11]. The MTHFR mutations, though traditionally considered low risk, may have potentiated immunothrombosis-a process wherein immune activation during severe infection triggers intravascular fibrin deposition, leading to ischemia and necrosis. The abrupt cessation of prophylactic anticoagulation postpartum may have further predisposed the patient to these thrombotic complications during systemic inflammatory stress [12].

The absence of positive blood cultures and atypical TSS rash pattern complicated the diagnosis, but the clinical picture of shock, fever, and multiorgan dysfunction fits well within the spectrum of TSS, possibly modified by the underlying thrombophilia. Even so-called "low-risk" genetic variants may assume greater clinical relevance in the presence of infection, inflammation, surgical stress, and disrupted anticoagulant therapy. Clinical vigilance, extended thromboprophylaxis, and early multidisciplinary intervention are critical to improving outcomes in such complex scenarios.

## Conclusions

This case describes an atypical presentation of postpartum TSS in a MTHFR C677T/A1298C compound heterozygote patient. Unlike the classical presentation, which features a diffuse erythematous rash, this patient developed purpuric and sharply demarcated skin necrosis-suggesting a thrombotic rather than purely inflammatory dermatologic process. Although coagulation abnormalities were only mild, her underlying genetic profile may have amplified the thromboinflammatory response, contributing to peripheral ischemia. This case underscores the importance of recognizing atypical manifestations of TSS, particularly in postpartum patients with genetic polymorphisms associated with an increased risk of thrombosis. It also reinforces the need for individualized thromboprophylaxis strategies in such patients, whose risk may be underestimated in the setting of infection, surgical stress, and hormonal shifts of the postpartum period.
